# Tracking of objective physical activity and physical fitness in Japanese children

**DOI:** 10.1186/s13104-019-4288-y

**Published:** 2019-05-07

**Authors:** Kensaku Sasayama, Minoru Adachi

**Affiliations:** 1 Faculty of Education, Okayama University of Science, 1-1, Ridai-cho, Kita-ku, Okayama, 700-0005 Japan; 2 Graduate School of Education, Okayama University, 3-1-1, Tsushima-naka, Kita-ku, Okayama, 700-8530 Japan

**Keywords:** Longitudinal, Youth, Moderate-to-vigorous physical activity, Fitness, Asian children

## Abstract

**Objective:**

The purpose of this study was to examine the tracking of objective physical activity and physical fitness from childhood to adolescence in Japanese children. The longitudinal study comprised 368 participants (aged 9–10 years) in 2008, and the study involved 134 participants (aged 13–14 years, a dropout rate of 63.6%) in 2011. After excluding participants with missing data, a total of 111 participants (46 boys and 65 girls) were available for study. Step counts and moderate-to-vigorous physical activity (MVPA) were measured using a uniaxial accelerometer. Physical fitness was assessed using the following tests: hand grip, sit-ups, sit and reach, side-to-side steps, 20-m shuttle run, 50-m dash, standing broad jump and ball throwing.

**Results:**

In boys, there was a significant correlation between objective physical activity and all physical fitness tests at baseline and follow-up. In girls, although there was no significant correlation between objective physical activity at baseline and follow-up, all physical fitness tests at baseline and follow-up were significantly correlated. In conclusion, moderate tracking was shown in objective physical activity of boys from childhood to adolescence. In addition, moderate to high tracking was shown in physical fitness of both sexes from childhood to adolescence.

## Introduction

Several studies have reported that physical activity in childhood is correlated to both physical and mental aspects [1, 2]. Therefore, the World Health Organization recommends that children aged 5–17 years age should accumulate at least 60 min of moderate-to-vigorous physical activity (MVPA) every day [3]. Similarly, the physical activity guidelines in the UK [4], USA [5] and Canada [6] also recommend this level of activity for similar age groups.

A review by Telema et al. [7] reported that physical activity tracks from childhood to adolescence and indicated that most previous studies used questionnaires to measure and assess physical activity. A questionnaire can easily evaluate physical activity and has the merit of low cost. However, previous studies [8] also reported that questionnaires overestimate physical activity compared to accelerometry. In addition, Chinapaw et al. [9] reported that the reliability of physical activity questionnaires for children (mean age 6–12 years) is lower than that for adolescents (mean age 12–18 years). Therefore, it appears to be important to examine the tracking of physical activity using objective methods such as an accelerometer.

Physical fitness as well as physical activity is reportedly related to physical and mental aspects [10]. Malina [11] examined the tracking of physical fitness (e.g. strength, flexibility and endurance) and reported that physical fitness tracks from childhood to adolescence at low to moderate levels. To the best of our knowledge, the tracking of objective physical activity and physical fitness from childhood to adolescence has neither been measured nor reported in Japanese children. Therefore, it is necessary to confirm the tracking of physical activity and physical fitness from childhood to adolescence to promote the importance of physical activity and physical fitness in this population.

The purpose of this study was to examine the tracking of objective physical activity and physical fitness from childhood to adolescence in Japanese children. We hypothesised that objective physical activity and physical fitness track from childhood to adolescence in Japanese children.

## Main text

### Methods

#### Study design and participants

This study was conducted in Ibara City within the Okayama Prefecture of Japan during 2008–2011 and encompassed all 13 public elementary schools within the city. The first study comprised 368 participants aged 9–10 years (90.6% within the city) in September 2008, and the follow-up study involved 134 participants (aged 13–14 years, with dropout rate of 63.6%) in September 2011. After excluding participants with missing data, a total of 111 participants (46 boys, 65 girls) were included in the study.

#### Physical activity

Total steps and MVPA were measured using a uniaxial accelerometer (Kenz Lifecorder EX (LC); Suzuken Co. Ltd, Nagoya, Japan). Kumahara et al. [12] have previously reported that this accelerometer samples acceleration at a rate of 32 samples/second and assesses values ranging from 0.06 to 1.94 g. The acceleration signal was filtered using an analogue bandpass filter and subsequently digitised. The maximum pulse over 4 s was measured as the acceleration value and classified into 11 activity levels (0, 0.5 and 1–9). The epoch length of the LC device is 2 min. Sasayama and Adachi [13] showed that the activity level detected using an LC device was significantly correlated with metabolic equivalents (METs) during walking and running in Japanese children (r = 0.883, p < 0.05). They confirmed that the activity level for MVPA (≥ 3 METs) is equivalent to a value of ≥ 5 as detected by the LC device; accordingly, the MVPA cut-off point was based on this finding [13]. Participants wore the LC device on their waists for 5 consecutive weekdays, at all times, excluding while sleeping, swimming, bathing or contact sports. Based on previous studies [14], accelerometer data were collected on at least 3 weekdays. A valid day was defined as at least 600 min of wear time during weekdays [15]. Non-wearing time was defined by at least 60 min of zero consecutive counts [16].

#### Physical fitness

Physical fitness was assessed using the new National Statistical Survey on Physical Fitness and Motor Ability by the Ministry of Education, Culture, Sports, Science and Technology (MEXT) in Japan (MEXT, 2000). The physical fitness test by MEXT was conducted as follows and test items included hand grip (muscle strength), sit-ups (abdominal strength and endurance), sit and reach (flexibility), side-to-side jump (agility), 20-m shuttle run (cardiorespiratory endurance), 50-m dash (speed), standing broad jump (explosive leg strength) and softball (9–10 years)/handball (13–14 years) throwing (explosive arm strength and throwing ability).

#### Anthropometry

Height (precision within 0.1 cm) and body weight (precision within 0.1 kg) were measured in light clothing without shoes. Body mass index (BMI) was calculated using the ratio of weight (kg) to height squared (m^2^).

#### Statistical analysis

Participant characteristics, physical activity and physical fitness variables were reported as mean ± standard deviations. Characteristic differences in baseline and follow-up were analysed using paired Student’s *t* test. The associations between the obtained variables were analysed using Spearman correlation coefficient. Values ranging from 0.00 to 0.29 indicate low correlation, from 0.30 to 0.59 indicate moderate correlation and from 0.60 to 1.00 indicate high correlation [11]. All analyses were performed using SPSS Statistics software version 24. Results were considered statistically significant at p < 0.05.

### Results

#### Characteristics of participants at baseline and follow-up

Characteristics of participants at baseline and follow-up are shown in Table 1. For both sexes, all variables of anthropometry, physical activity and physical fitness showed significant differences between baseline and follow-up.

**Table 1:** Participants of characteristics

	Boys (n = 46)			Girls (n = 65)		
	Baseline (2008)	Follow-up (2011)	p value	Baseline (2008)	Follow-up (2011)	p value
Age (years)	9.2 ± 0.4	13.2 ± 0.4	< 0.001	9.1 ± 0.3	13.1 ± 0.3	< 0.001
Height (cm)	134.1 ± 5.8	153.1 ± 8.2	< 0.001	135.1 ± 5.4	152.0 ± 4.1	< 0.001
Weight (kg)	30.7 ± 7.0	44.4 ± 10.2	< 0.001	29.8 ± 4.5	43.9 ± 6.3	< 0.001
BMI (kg/m^2^)	16.9 ± 2.9	18.8 ± 3.0	< 0.001	16.2 ± 1.7	19.0 ± 2.4	< 0.001
Total steps (steps/day)	18387.7 ± 3876.4	12188.5 ± 4872.9	< 0.001	14627.3 ± 3340.2	11863.4 ± 3153.3	< 0.001
MVPA (min/day)	60.6 ± 18.5	37.3 ± 22.0	< 0.001	39.9 ± 13.5	32.8 ± 14.3	0.004
Hand grip (kg)	14.9 ± 3.0	23.4 ± 6.9	< 0.001	14.0 ± 3.3	21.8 ± 4.6	< 0.001
Sit-ups (count)	19.3 ± 5.7	24.2 ± 6.2	< 0.001	14.6 ± 3.9	18.7 ± 3.9	< 0.001
Sit and reach (cm)	36.5 ± 8.7	40.2 ± 8.5	0.015	37.2 ± 7.7	42.4 ± 7.1	< 0.001
Side-to-side jump (count)	40.5 ± 7.0	48.9 ± 5.8	< 0.001	37.7 ± 5.6	43.4 ± 3.9	< 0.001
20-m shuttle run (count)	45.7 ± 16.8	68.1 ± 20.9	< 0.001	29.9 ± 11.6	46.0 ± 12.8	< 0.001
50-m dash (s)	9.4 ± 0.7	9.0 ± 0.9	< 0.001	9.7 ± 0.7	9.3 ± 0.7	< 0.001
Standing broad jump (cm)	147.6 ± 14.7	174.7 ± 20.7	< 0.001	141.1 ± 16.1	156.9 ± 25.4	< 0.001
Ball throwing (m)	24.6 ± 6.8	19.6 ± 4.5	< 0.001	13.7 ± 5.1	11.4 ± 3.4	< 0.001

Values are means ± standard deviations

BMI: Body mass index; MVPA: moderate-to-vigorous physical activity

p < 0.05 for different baseline and follow-up

#### Tracking of physical activity and physical fitness

Tracking of physical activity and physical fitness expressed as Spearman correlation coefficients are shown in Table 2. In boys, there was a significant correlation between total steps, MVPA, hand grip, sit-ups, sit and reach, side-to-side jump, 20-m shuttle run, 50-m dash, standing broad jump and ball throwing at baseline and follow-up. In girls, although there was no significant correlation between total steps and MVPA at baseline and follow-up, hand grip, sit-ups, sit and reach, side-to-side jump, 20-m shuttle run, 50-m dash, standing broad jump and ball throwing at baseline and follow-up were significantly correlated.

**Table 2:** Tracking of physical activity and physical fitness expressed as Spearman correlation coefficients

	Boys (n = 46)		Girls (n = 65)	
	r	p value	r	p value
Physical activity				
Total steps (steps/day)	0.319	0.031	0.178	0.156
MVPA (min/day)	0.352	0.017	0.198	0.114
Physical fitness				
Hand grip (kg)	0.520	< 0.001	0.680	< 0.001
Sit-ups (count)	0.588	< 0.001	0.575	< 0.001
Sit and reach (cm)	0.341	0.020	0.303	0.014
Side-to-side jump (count)	0.470	< 0.001	0.444	< 0.001
20-m shuttle run (count)	0.618	< 0.001	0.513	< 0.001
50-m dash (s)	0.736	< 0.001	0.734	< 0.001
Standing broad jump (cm)	0.548	< 0.001	0.602	< 0.001
Ball throwing (m)	0.647	< 0.001	0.549	< 0.001

MVPA: Moderate-to-vigorous physical activity

### Discussion

The objective evaluation of physical activity by an accelerometer was a notable strength of the study. Adachi et al. [17] have previously reported that the total energy expenditure assessed using the doubly-labelled water method was significantly correlated with total steps and activity level detected using an accelerometer in Japanese children. In Japanese children aged 9–13 years, we found moderate tracking of physical activity (only boys) and moderate to high tracking of physical fitness (both boys and girls).

Previous studies [18–21] measuring total physical activity using pedometers or accelerometers have reported that total physical activity tracks from childhood to adolescence at low to moderate levels. Although these studies differ from the current study in terms of the ages of participants, follow-up period and pedometer or accelerometer use, the present study also showed moderate tracking for total steps in boys (Table 2). On the other hand, in our study, tracking was not confirmed for total steps in girls (Table 2). In some previous studies, tracking of total physical activity in both boys and girls reported similar results [20] but some studies [19, 21] showed that high tracking of total physical activity was confirmed in boys compared to girls. Therefore, there are possibilities of gender differences in terms of tracking. This gender difference may be influenced by the fact that total physical activity in girls is lower than that of boys from childhood to adolescence. Indeed, Wolff-Hughes et al. [22] reported that total physical activity in boys was higher than that in girls, regardless of age (6–19 years). Wolff-Hughes et al. [22] also reported that total physical activity in girls tends to sharply decrease at adolescence compared to boys. This phenomenon may influenced tracking from childhood to adolescence.

Previous studies [18, 23–26] have reported that MVPA using accelerometer tracks from childhood to adolescence at low to moderate levels. We confirmed that MVPA tracks moderately only in boys from childhood to adolescence (Table 2). It has been reported that MVPA tracking is more robust in girls than in boys [24]. On the other hand, another report showed that MVPA tracking was similar for boys and girls [25]. Therefore, for MVPA as well as total steps, it is necessary to confirm whether there is gender difference in tracking.

Regarding the tracking of physical fitness, it has been reported elsewhere [11] that physical fitness, such as muscular strength and endurance, power or explosive strength, running speed, agility, cardiovascular fitness track at low to moderate levels from childhood to adolescence. In our study, low to high tracking was confirmed in various physical fitness components (Table 2). Therefore, it was suggested that various physical fitness components track from childhood to adolescence in Japanese children.

### Conclusion

Our results suggest that objective physical activity in Japanese boys track moderately from childhood to adolescence. In addition, for both Japanese boys and girls, physical fitness track moderately to highly from childhood to adolescence.

## Limitations

Our study has several limitations. First, this study comprised a small sample size, had a short tracking term and was conducted only in the Okayama Prefecture in Japan. Since the study was conducted only in the Okayama Prefecture in Japan, its results cannot be applied to other populations or regions. Second, there were no controls for maturity related to physical fitness. Further studies are required with larger sample sizes and including participants from other populations. In addition, it is necessary to examine the maturity level.

## Data Availability

Please contact author for data requests.

## Abbreviations

MVPA: moderate-to-vigorous physical activity
LC: lifecorder
METs: metabolic equivalents
MEXT: Ministry of Education, Culture, Sports, Science and Technology
BMI: body mass index
